# Artificial intelligence–assisted detection of challenging ischemic stroke on diffusion-weighted imaging: a reader study

**DOI:** 10.3389/fneur.2026.1766199

**Published:** 2026-05-21

**Authors:** Younbeom Jeong, Wi-Sun Ryu, Beom Joon Kim, Byung Se Choi, Jae Hyoung Kim, Leonard Sunwoo

**Affiliations:** 1 Department of Radiology, Seoul National University Bundang Hospital, Seoul National University College of Medicine, Seongnam, Republic of Korea; 2 Artificial Intelligence Research Center, JLK Inc., Seoul, Republic of Korea; 3 Department of Neurology, Seoul National University Bundang Hospital, Seoul National University College of Medicine, Seongnam, Republic of Korea; 4 Center for Artificial Intelligence in Healthcare, Seoul National University Bundang Hospital, Seongnam, Republic of Korea

**Keywords:** AI assisted diagnosis, artificial intelligence, diffusion magnetic resonance imaging, ischemic stroke, radiologists, reader study

## Abstract

**Background and purpose:**

To evaluate the effect of artificial intelligence (AI) assistance on the diagnostic performance of human readers for detecting challenging acute ischemic stroke (AIS) lesions on diffusion-weighted MRI.

**Methods:**

This retrospective, single-center, randomized crossover study included 3,986 patients (mean age, 68 ± 14 years; 2,383 men) who underwent initial and follow-up diffusion-weighted imaging (DWI) between February 2017 and November 2021. From this cohort, 250 cases (130 challenging AIS, 120 controls) were selected for a multi-reader performance study. Five readers interpreted cases with and without AI assistance. Diagnostic performance was evaluated using the area under the receiver operating characteristic curve (AUC), sensitivity, specificity, and Dice similarity coefficient (DSC).

**Results:**

AI achieved a sensitivity of 96.0% and identified 79.6% (43 of 54) of false-negative stroke cases from clinical reports. AI-assisted reading significantly improved AUC from 0.85 (95% CI: 0.82–0.90) to 0.93 (95% CI: 0.90–0.95; *p* < 0.01), pooled sensitivity from 74.6% (95% CI: 69.8–79.4) to 90.6% (95% CI: 87.4–93.7; *p* < 0.01), and lesion segmentation accuracy (DSC) from 0.523 to 0.742 (*p* < 0.01). Specificity slightly decreased from 88.8% (95% CI: 85.1–92.3) to 84.0% (95% CI: 78.5–89.5; *p* = 0.05). Reader confidence also improved with AI support, especially in challenging cases.

**Conclusions:**

AI assistance significantly improved diagnostic performance and lesion segmentation accuracy in detecting small and hyperacute AIS lesions on DWI.

## Introduction

Diffusion-weighted imaging (DWI) is highly sensitive for diagnosing acute ischemic stroke (AIS) (1–3). Early ischemic changes can be visualized on DWI within 2 h after symptom onset (1), and animal models suggest detectable abnormalities as early as 3–5 min after onset (4, 5), However, hyperacute stroke or small AIS lesions—particularly in the brainstem—are often overlooked in clinical practice (6–9). Prior studies have demonstrated that such lesions are prone to false-negative findings on standard DWI, (7, 9) and Makin et al. showed that lesions as small as 0.045 ml may be missed by standard protocols (10). Missing these diagnostically challenging infarcts may delay treatment and hinder timely secondary prevention.

The clinical impact of missing small strokes is substantial. Even minor infarcts are associated with increased risk of recurrent ischemic events, progression to larger infarcts, and persistent neurological deficits (9, 10). Importantly, the clinical burden of small lesions often depends more on their location than on their absolute volume; for example, a 0.5 ml lesion in the brainstem can cause disabling syndromes such as ataxic hemiparesis or internuclear ophthalmoplegia despite a low National Institutes of Health Stroke Scale (NIHSS) score (9). Current European Stroke Organization guidelines also highlight the importance of recognizing such subtle lesions to enable appropriate secondary prevention (11).

Despite advances in neuroimaging, stroke missed diagnosis remains a significant clinical problem, particularly in the emergency department. A meta-analysis reported a false-negative rate of 8.7% for cerebrovascular events, suggesting that over 100,000 strokes and transient ischemic attacks (TIAs) are missed annually in the United States alone (12). Missed strokes can lead to severe disability, increased mortality, and long-term economic burdens, particularly in younger patients (13, 14). Among the 45,000–75,000 strokes missed in dizziness patients annually, 15,000–25,000 may have suffered preventable major strokes (15). These findings highlight the need to improve the detection of small and hyperacute AIS to prevent avoidable harm.

Recently, artificial intelligence (AI)-based tools have been introduced to aid in AIS lesion detection on DWI (16, 17). Among them, JLK-DWI (JLK Inc.), a deep-learning-based solution, has shown high sensitivity (98.1%) for small (< 10 ml) AIS lesions (6). However, in clinical practice, most AI solutions currently serve as an adjunct rather than a stand-alone diagnostic tool, aiding human experts in making the final decisions (18). Thus, the ultimate value of AI depends on effective human-AI collaboration (19). If radiologists accept AI-driven false-positives or reject true-positive lesions, performance may worsen. Conversely, if they accept true-positive detections initially missed by themselves and successfully disregard false-positive AI suggestions, diagnostic performance can improve. Despite these considerations, the impact of AI on AIS detection, particularly in cases where infarcts are small, located in critical regions, or occur in hyperacute settings—situations where detection is most challenging—remains insufficiently explored.

In this study, we conducted a reader performance study to determine whether JLK-DWI assistance improves diagnostic accuracy in challenging AIS cases, defined by strict volume and time-based criteria. We also compared radiology reports of DWI to AI-generated outputs to evaluate potential benefits in clinical decision-making.

## Methods

### Study design and ethics

This retrospective, single-center, randomized crossover study was conducted at Seoul National University Bundang Hospital, with approval from the institutional review board (IRB No. B-2405-900-102). The requirement for written informed consent was waived for both patients and human readers.

### Patient cohort and ground truth definition

**Figure 1:**
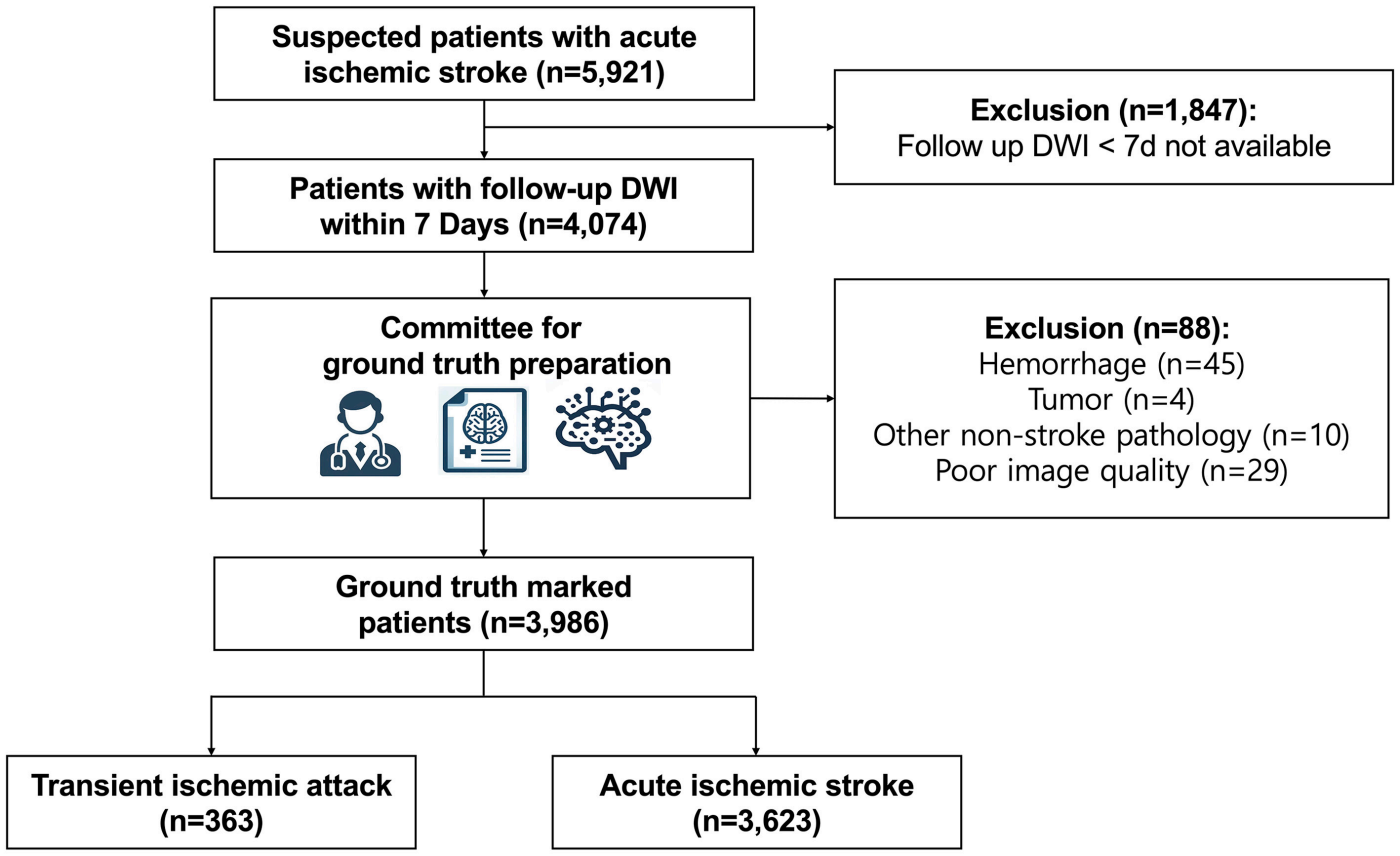
Patient inclusion flow chart. Flowchart depicting patient inclusion and exclusion criteria for the study cohort based on diffusion-weighted imaging (DWI) results.

Initially, 5,921 consecutive patients with suspected AIS who visited Seoul National University Bundang Hospital from February 2017 to November 2021 were selected from the registry of the Clinical Research Collaboration for Stroke in Korea, a government-funded national stroke registry project (20). From the cohort, 4,074 patients who underwent both initial and follow-up DWI within 7 days were included. After excluding 88 patients due to non-ischemic diagnoses (e.g., hemorrhagic stroke, tumors) or poor image quality, 3,986 patients were finally enrolled (Figure 1).

Ground truth at the case level (AIS or TIA) was determined by an expert panel comprising one stroke neurologist (B.J.K.) with 17 years of experience and two neuroradiologists (L.S. and J.H.K.) with 15 and 39 years of experience, respectively. The panel reviewed clinical records, radiology reports, and initial and follow-up DWI scans to determine the final diagnosis. AIS was defined as a persistent hyperintense lesion on initial DWI with corresponding apparent diffusion coefficient (ADC) reduction, demonstrating chronological changes on follow-up DWI, and clinically correlating with stroke symptoms. TIA was defined as the absence of visible lesions on both initial and follow-up DWI, with a clinical course consistent with TIA. For lesion-level analyses, ground truth segmentation masks were created on DWI maps for AIS cases through manual annotation. Each case was independently assessed by the three reviewers, and discrepancies were resolved through consensus discussions. If consensus was not reached, the final decision was made by the senior neuroradiologist (J.H.K.).

### MRI examination

DWI was performed using a 1.5-T (Intera, Philips Healthcare; and Magnetom Amira, Siemens Healthineers) or 3.0-T scanner (Achieva, Ingenia, or Elition, Philips Healthcare) with an 8- or 32-channel head coil. Imaging parameters were standardized across scanners: b = 1,000 s/mm^2^, repetition time = 2,400–15,000 ms, echo time = 44–140 ms, slice thickness = 5 mm with a 1 mm interslice gap (slice spacing = 6 mm), and field of view = 200–274 mm^2^. The acquisition matrix ranged from 128 × 128 to 256 × 256, yielding an in-plane resolution of approximately 0.9–1.3 mm. ADC maps were generated using monoexponential fitting.

### AI processing and classification

All DWIs were analyzed using commercially available AI-based software (JLK-DWI). The software employs a U-Net-based algorithm to assign a probability of high signal intensity to each voxel on DWI and generates lesion masks using a 0.5 probability threshold (16). Lesion volumes are then calculated using voxel spacing.

For each case, the AI result was compared with the ground truth to classify the result as true positive, false positive (FP), true negative, or false negative (FN).

### Reader performance study with challenging cases

To evaluate human-AI interactions, 130 challenging cases, 40 positive control, and 80 negative control cases were randomly selected from the cohort, resulting in a total of 250 cases. In this study, “challenging” was used in a narrow, operational sense to capture lesions expected to be difficult to detect visually on DWI/ADC. We defined challenging cases as those with either: (1) an infarct volume < 0.5 ml in the posterior circulation, or (2) an infarct volume < 1.0 ml in the anterior circulation within 3 h of symptom onset. Positive control cases were selected from those with infarction volumes of 1–6 ml to avoid excessively large lesions. Negative control cases were chosen from DWI-negative TIA cases. To minimize selection, cases included in the reader performance study were sampled so that the distribution of standalone AI performance (e.g., sensitivity and specificity) in this subset closely reflected that of the entire cohort.

Five readers participated: two 1st-year radiology residents (reader 1, reader 2), one 1st-year neurology resident (reader 3), one 3rd-year neurology resident (reader 4), and one neuroradiologist with 4 years of experience (reader 5). None of the participating readers had prior experience with the AI software, and each completed a standardized training session before commencing the reader study.

**Figure 2:**
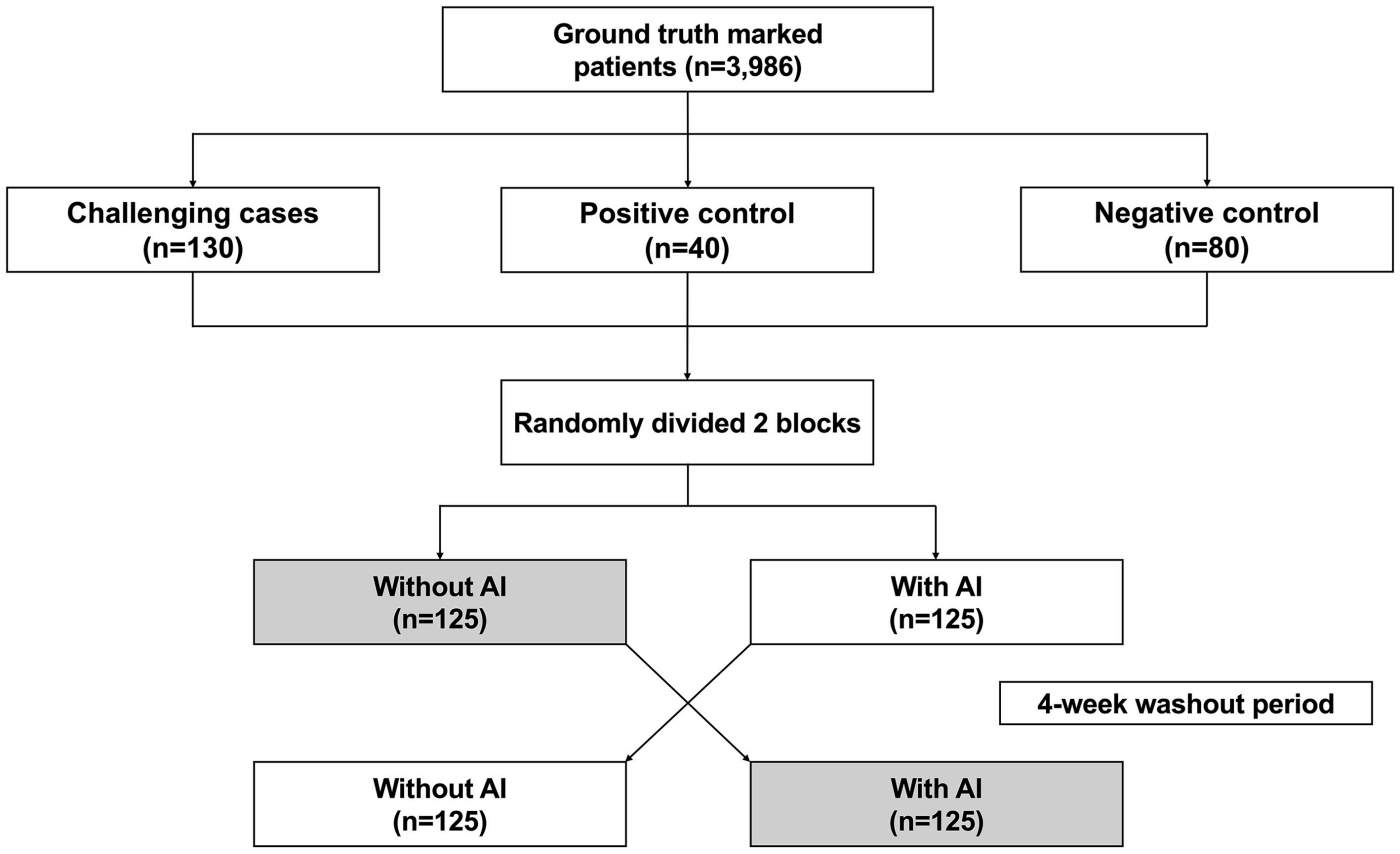
Reader study flow. Flowchart illustrating the crossover design of the multi-reader study, including random assignment to AI-assisted or unassisted reading, followed by a 4-week washout period and reversed assignment.

Each reader interpreted DWI + ADC maps in two sessions (AI-assisted vs. non-assisted), separated by a 4-week washout, with randomized case order and AI status (Figure 2). Each reader was asked to evaluate the presence of stroke by providing both a binary judgment (positive/negative) and a 5 point confidence score (0 = definitely not AIS; 4 = definitely AIS). Lesion segmentation was performed only for cases judged as positive; when multiple acute infarcts were present, all were annotated. During AI-assisted sessions, the AI-generated lesion masks were displayed for review and optional adjustment. Confidence scores were analyzed separately to assess changes in diagnostic confidence but did not affect binary classification. Readers were blinded to the study hypothesis, AI performance, and case distribution, as well as to all clinical information (including presenting symptoms, neurologic examination findings, and onset-to-imaging time). They were informed only that the cases were selected for stroke assessment, and their task was to evaluate DWI and ADC maps for acute ischemic lesions.

### AI false positive analysis

Two neuroradiologists (L.S., J.H.K.) reviewed FP lesions by AI, classifying their anatomical locations (e.g., cerebral cortex, internal capsule, periventricular or subcortical white matter, basal ganglia, cerebellum, pons, midbrain, and medulla) and underlying mechanisms. The mechanisms were classified as follows: (1) susceptibility artifacts from normal anatomy, (2) accentuated T2 hyperintensity of normal anatomy, (3) accentuated T2 hyperintensity from pathological lesions (e.g., small vessel disease), and (4) susceptibility artifacts from adjacent pathologies (e.g., microbleeds).

### Statistical analysis

The diagnostic performance of AI-assisted and non-assisted readings was compared using area under the receiver operating characteristic curve (AUC), sensitivity, specificity, and Dice similarity coefficient (DSC). The Obuchowski-Rockette method and the MRMCaov library (R package; version, 4.3.0; R Project for Statistical Computing) were used for multi-reader, multi-case analyses (21). Paired t-tests and additional analyses were performed using MedCalc software (version 23.0.2; MedCalc Software Ltd, Mariakerke, Belgium). A *p*-value < 0.05 was considered statistically significant. No further multiple comparison corrections were required for multi-reader, multi-case analyses.

## Results

### Patient characteristics

Table 1 summarizes patient demographics and clinical features, while Figure 1 details the selection process. Among the 3,986 patients (mean age, 68 ± 14 years; 2,383 men, 1,603 women), 3,623 (90.9%) had AIS and 363 (9.1%) had TIA. Of the AIS patients, 2,202 (60.8%) had only anterior circulation strokes, 1,192 (32.9%) had only posterior circulation strokes, and 229 (6.3%) had involvement in both territories. The median onset-to-DWI time was 9 h (IQR 3–31 h). The median DWI infarction volume was 0.99 mL (IQR 0.29–5.96; full range, 0.03–430), and 1,294 patients (35.7%) had small infarcts ( ≤ 0.5 ml). Stroke risk factors such as obesity, hypertension, diabetes, hyperlipidemia, smoking, and atrial fibrillation differed significantly between AIS and TIA groups (all *p* < 0.05).

**Table 1:** Patient demographics and clinical characteristics.

Characteristic	Total	AIS	TIA	*P*-value
**No. of patients** ^*^	3,986	3,623 [90.9]	363 [9.1]	
**Male (%)** ^*^	2,383 [59.8]	2,210 [61.0]	173 [47.7]	**< 0.01**
**Patient age** ^**†**^	67.94 (13.78)	68.46 (13.53)	62.75 (15.19)	**< 0.01**
**Stroke type**				
**Anterior circulation only** ^*^		2,202 [60.8]		
**Posterior circulation only** ^*^		1,192 [32.9]		
**Both circulations** ^*^		229 [6.3]		
**NIHSS at admission** ^**†**^	5.16 (5.87)	5.56 (5.97)	1.18 (2.18)	**< 0.01**
**Infarct volume (ml)** ^**‡**^		0.99 (0.29–5.96)		
**Obesity** ^*^	1,410 [35.4]	1,256 [34.7]	154 [42.4]	**< 0.01**
**Hx. of TIA** ^*^	123 [3.1]	99 [2.7]	24 [6.6]	**< 0.01**
**Hx. of stroke** ^*^	807 [20.2]	736 [20.3]	71 [19.6]	0.79
**Hx. of PAD** ^*^	26 [0.7]	24 [0.7]	2 [0.6]	1.00
**Hx. of CHD** ^*^	409 [10.3]	379 [10.5]	30 [8.3]	0.22
**HTN** ^*^	2,734 [68.6]	2,529 [69.8]	205 [56.5]	**< 0.01**
**DM** ^*^	1,289 [32.3]	1,206 [33.3]	83 [22.9]	**< 0.01**
**HL** ^*^	1,664 [41.7]	1,494 [41.2]	170 [46.8]	0.05
**Smoking** ^*^	1,327 [33.3]	1,234 [34.1]	93 [25.6]	**< 0.01**
**Afib** ^*^	733 [18.4]	707 [19.5]	26 [7.2]	**< 0.01**

AIS, acute ischemic stroke; TIA, transient ischemic attack; Hx, history; PAD, peripheral arterial disease; CHD, coronary heart disease; HTN, hypertension; DM, diabetes mellitus; HL, hyperlipidemia; Afib, atrial fibrillation. ^*^Proportion percentage in square brackets. ^†^Data are means and data in parentheses are SDs. ^‡^Volume of infarction on diffusion-weighted imaging, expressed as the median and interquartile range (IQR). Bold *P*-values indicate statistical significance (*P* < 0.05).

In our 932 challenging cases, 686 (73.6%) had NIHSS scores of 0–4 (minor stroke), while 246 (26.4%) had NIHSS ≥5 (Supplementary Table S1), representing moderate-to-severe strokes (22). The proportion of patients with modified Rankin Scale score ≥3 increased from 5.5% pre-stroke to 18.1% at 3 months (Supplementary Figure S1).

### Performance of standalone AI and initial radiology report

For all 3,986 cases, the standalone AI demonstrated a sensitivity of 96.0%, specificity of 51.8%, positive predictive value (PPV) of 95.2%, and negative predictive value (NPV) of 56.1%. Among the 363 TIA cases, the AI produced false-positive lesion markings in 175 cases (48.2%), consistent with the modest specificity; the anatomic distribution and patterns of these false positives are described in the following section. In comparison, radiology reports yielded a sensitivity of 98.4%, specificity of 98.6%, PPV of 99.8%, and NPV of 90.4% (Table 2).

**Table 2:** Diagnostic performance of AI standalone and radiology reports.

Metric	Initial radiology report^*^	AI standalone^*^	Initial radiology report in reader study cohort^†^	AI standalone in reader study cohort^†^
**Sensitivity (%)**	98.4 (98.0–98.8)	96.0 (95.2–96.5)	80.1 (74.2–85.6)	94.1 (89.5–96.8)
**Specificity (%)**	98.6 (97.2–99.3)	51.8 (46.7–56.9)	96.9 (89.5–99.2)	50.0 (39.3–60.1)
**Positive predictive value (%)**	99.8 (99.6–99.9)	95.2 (94.5–95.9)	98.7 (95.3–99.6)	80.0 (73.9–85.0)
**Negative predictive value (%)**	90.4 (87.6–92.5)	56.1 (50.8–61.3)	63.6 (53.8–72.4)	80.0 (67.0–88.8)

Data in parentheses are 95% confidence intervals. AI, artificial intelligence. ^*^Performance in the entire cohort. ^†^Performance calculated for the 250 cases included in the reader study.

Among the 54 FN cases in the radiology reports, half were in the anterior circulation and half in the posterior circulation. Notably, 70.4% (38 of 54; 11 in the anterior and 27 in the posterior circulation territory) of FN cases met the criteria for challenging cases. AI correctly identified 79.6% (43 of 54) of the FN cases in the radiology reports, including all 16 non-challenging cases and 27 of 38 challenging cases (8 in the anterior and 19 in the posterior circulation territory).

In a subgroup analysis by TOAST stroke etiology, standalone AI sensitivity remained consistently high across the three most common categories: LAA, 96.7%; CE, 97.1%; and SVO, 96.3%, with no significant between-group difference (*p* = 0.740) (Table S2).

In a subgroup analysis stratified by MRI field strength (1.5-T, *n* = 938; 3.0-T, *n* = 3,046), standalone AI sensitivity remained high in both subgroups [1.5-T: 96.9% (95% CI: 95.5–97.9); 3.0-T: 95.6% (95% CI: 94.8–96.3)]. Specificity was numerically lower at 3.0-T [1.5-T: 59.4% (95% CI: 47.1–70.5) vs. 3.0-T: 50.2% (95% CI: 44.5–55.8)], but the difference was not statistically significant (*p* = 0.215).

### Reader performance assessments

Among the 3,623 AIS cases, 932 (25.7%) were classified as challenging cases, and 130 of these were randomly chosen for the reader study. AI standalone performance in the reader cases was adjusted to align with that of the entire cohort, resulting in sensitivity of 94.1%, specificity of 50.0%, PPV of 80.0%, and NPV of 80.0%, respectively (Table 2).

**Figure 3:**
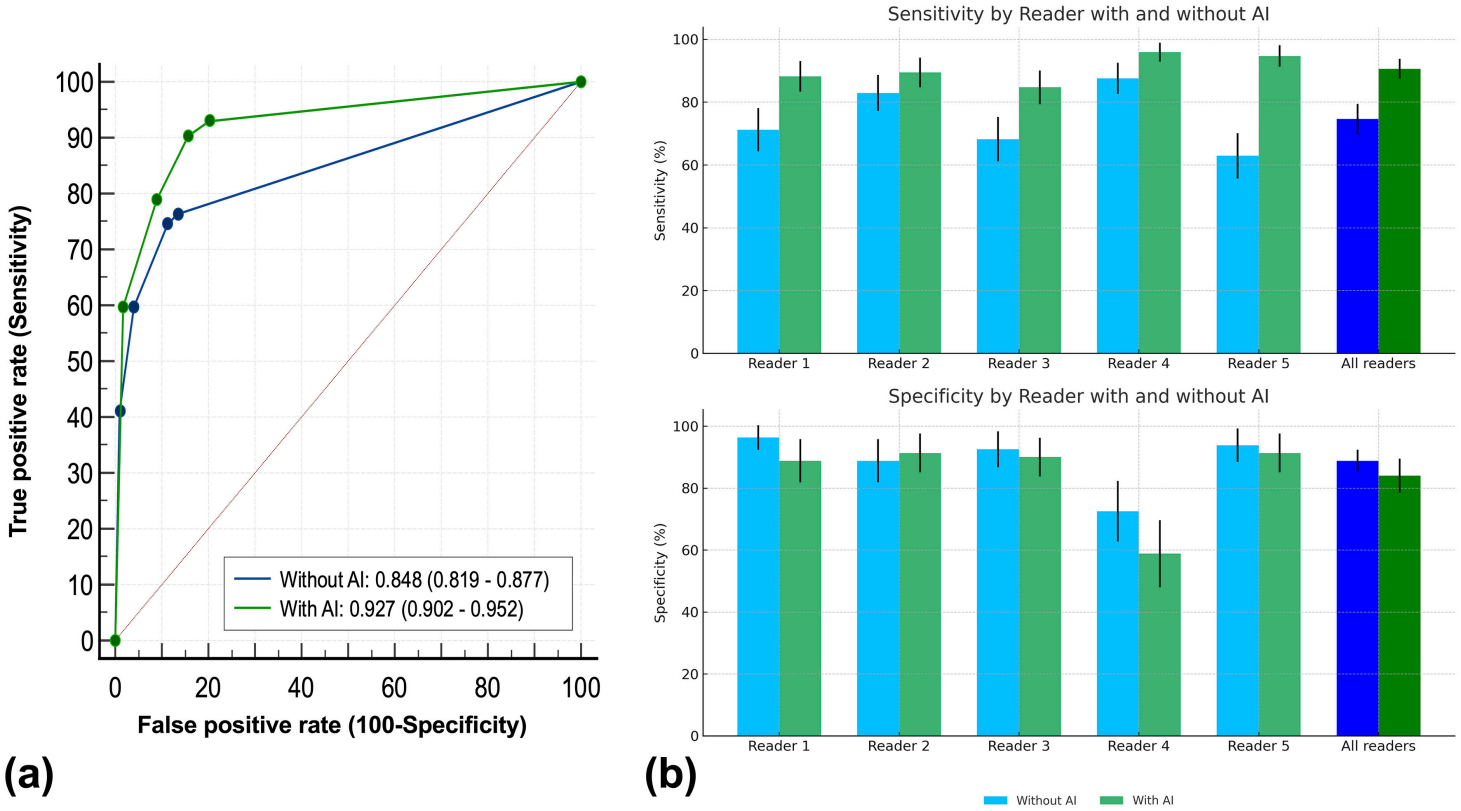
Comparison of diagnostic performance with and without AI assistance. **(a)** Receiver operating characteristic (ROC) curve analysis comparing diagnostic accuracy with AI (green) and without AI (blue). The area under the curve (AUC) is higher with AI assistance (0.927, 95% CI: 0.902–0.952) compared to without AI (0.848, 95% CI: 0.819–0.877). **(b)** Sensitivity and specificity by individual readers with and without AI. Sensitivity **(top)** and specificity **(bottom)** are shown as bar graphs for each reader and all readers, demonstrating improved sensitivity with AI across most readers, while specificity remains relatively stable. Error bars represent 95% confidence intervals.

Without AI assistance, the mean AUC for all readers was 0.85 (95% CI: 0.82–0.90). Reading with AI assistance improved the overall AUC to 0.93 (95% CI: 0.90–0.95; *p* < 0.01) (Table 3). Pooled sensitivity significantly increased from 74.6% (127 of 170) to 90.6% (154 of 170), and DSC improved from 0.523 to 0.742 (both *p* < 0.01). Pooled specificity showed a slight decrease from 88.8% (71 of 80) to 84.0% (67 of 80) (*p* = 0.05) (Figure 3).

**Table 3:** Reader study diagnostic performance.

Reader	With AI	Without AI	Difference^*^	*P*-value
AUC				
All readers	**0.927 (0.903–0.952)**	**0.848 (0.819–0.877)**	**0.0789**	**< 0.01**
Reader 1	0.938 (0.901–0.965)	0.854 (0.805–0.896)	0.0838	< 0.01
Reader 2	0.937 (0.899–0.963)	0.896 (0.851–0.931)	0.0405	0.04
Reader 3	0.884 (0.838–0.921)	0.815 (0.761–0.861)	0.0691	< 0.01
Reader 4	0.925 (0.885–0.954)	0.884 (0.838–0.921)	0.0407	0.04
15.6-7.4,-1.3498pt Reader 5	0.951 (0.916–0.974)	0.791 (0.735–0.840)	0.160	< 0.01
Sensitivity (%)				
All readers	**90.6 (87.4–93.7)**	**74.6 (69.8–79.4)**	**16.0**	**< 0.01**
Reader 1	88.2 (83.4–93.1)	71.2 (64.3–78.0)	17.1	< 0.01
Reader 2	89.4 (84.7–94.1)	82.9 (77.2–88.6)	6.5	< 0.01
Reader 3	84.7 (79.3–90.1)	68.2 (61.2–75.3)	16.5	< 0.01
Reader 4	95.9 (92.9–98.9)	87.6 (82.7–92.6)	8.2	< 0.01
15.6-7.4,-1.3498pt Reader 5	94.7 (91.3–98.1)	62.9 (55.7–70.2)	31.8	< 0.01
Specificity (%)				
All readers	**84.0 (78.5–89.5)**	**88.8 (85.1–92.3)**	**−4.75**	**0.05**
Reader 1	88.8 (81.8–95.7)	96.3 (92.1–100)	−7.50	0.03
Reader 2	91.3 (85.0–97.5)	88.8 (81.8–95.7)	2.50	0.53
Reader 3	90.0 (85.0–97.5)	92.5 (86.7–98.3)	−2.50	0.48
Reader 4	58.8 (47.9–69.6)	72.5 (62.7–82.3)	−13.8	0.44
15.6-7.4,-1.3498pt Reader 5	91.3 (85.0–97.5)	93.8 (88.4–99.1)	−2.50	0.42
DSC				
All readers	**0.742 (0.722–0.761)**	**0.523 (0.545–0.601)**	**0.219**	**< 0.01**
Reader 1	0.791 (0.751–0.833)	0.614 (0.567–0.661)	0.178	< 0.01
Reader 2	0.774 (0.732–0.816)	0.409 (0.360–0.459)	0.365	< 0.01
Reader 3	0.761 (0.717–0.806)	0.540 (0.491–0.589)	0.221	< 0.01
Reader 4	0.554 (0.509–0.599)	0.537 (0.492–0.582)	0.016	0.58
Reader 5	0.828 (0.791–0.865)	0.515 (0.465–0.564)	0.313	< 0.01

Data in parentheses are 95% confidence intervals. AUC, area under the receiver operating characteristic curve; AI, artificial intelligence; DSC, Dice similarity coefficient. Bold text highlights overall performance across all readers. ^*^Difference = diagnostic performance with AI—diagnostic performance without AI.

**Figure 4:**
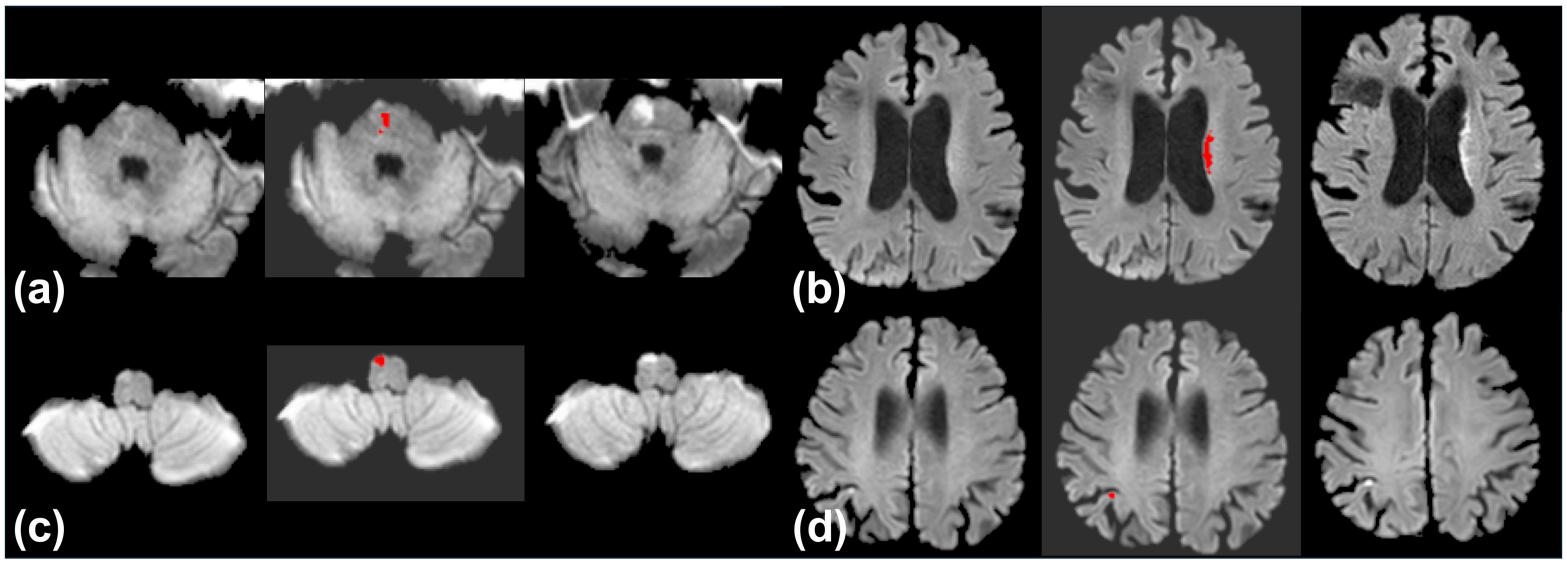
Representative cases of acute ischemic stroke lesions that were initially missed by most or all reviewers but correctly identified with AI assistance. **(a)** Pontine infarction not detected by any reviewer on initial MRI (15 h from last known well); identified by three reviewers with AI assistance. Follow-up MRI at 2 days confirmed the lesion. **(b)** Corona radiata infarction undetected by all reviewers on initial MRI (2 h); correctly identified by three with AI support. Confirmed on follow-up MRI at 4 days. **(c)** Medial medullary infarction not recognized by any reviewer at baseline MRI (3 h); identified by three with AI assistance. Confirmed on follow-up at 2 days. **(d)** Subtle parietal cortex infarction missed by four reviewers on initial MRI (2 h); correctly detected with AI. Follow-up at 4 days confirmed the lesion.

All individual readers improved their AUC, sensitivity, and DSC with AI assistance. The largest improvement in AUC occurred for Reader 5 (0.79 to 0.95), accompanied by a sensitivity increase from 62.9% to 94.7%. The most notable specificity drop was for Reader 4 (from 72.5 to 58.8%). Confidence scores for challenging cases increased with AI support (Supplementary Figure S2). Representative examples of challenging cases are shown in Figure 4.

### AI false positive distribution

**Figure 5:**
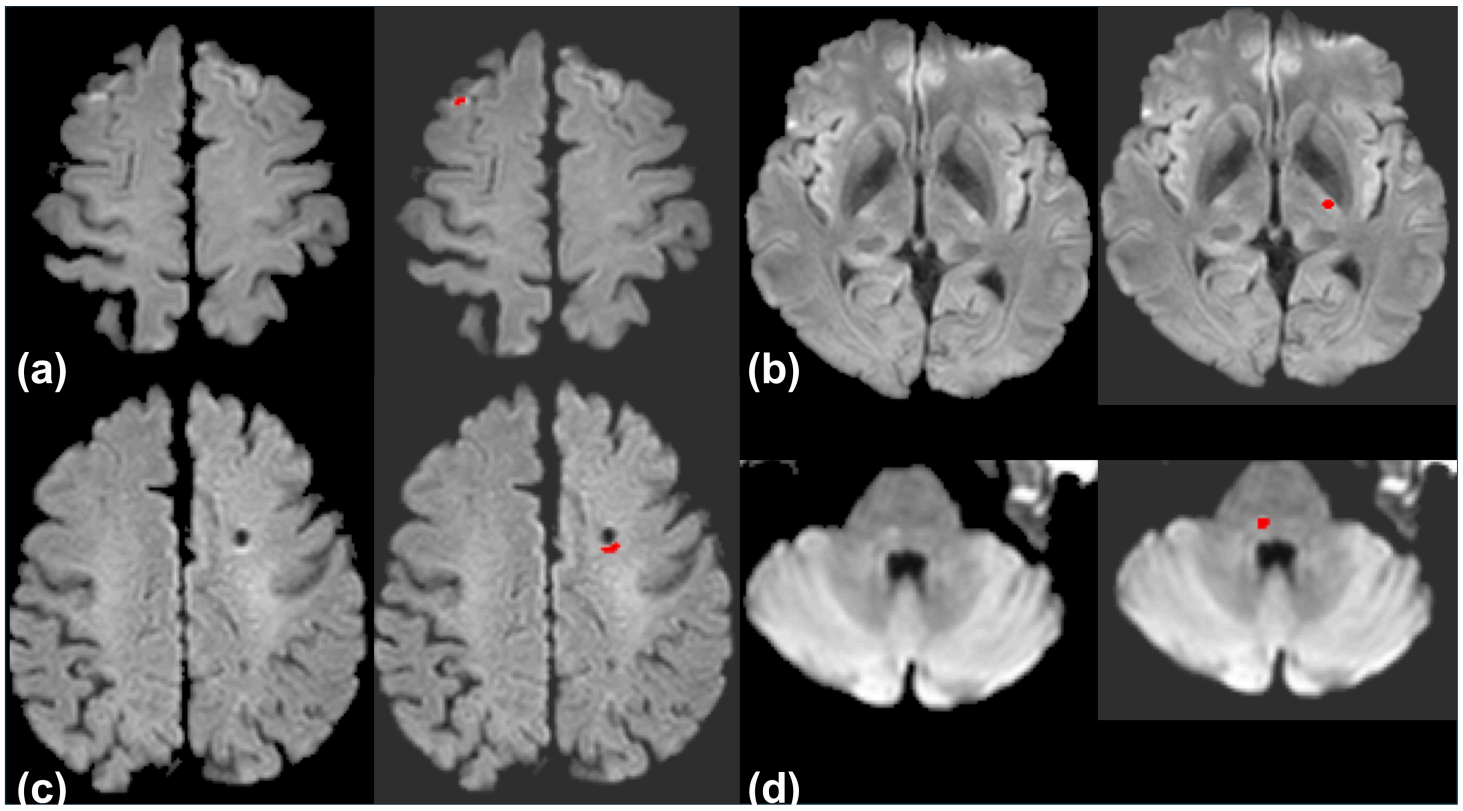
Representative cases of common false-positive lesions detected by the AI model. **(a)** Right frontal cortex: susceptibility artifact at the brain-skull interface. **(b)** Posterior limb of the left internal capsule: accentuated T2-weighted signal of normal anatomy. **(c)** Left frontal white matter: susceptibility artifact from adjacent microbleeds. **(d)** Right pons (medial lemniscus): hyperintensity related to small vessel disease.

Common locations of FP detections by AI are summarized in Table 4. The posterior limb of the internal capsule was a frequent site, likely due to intrinsic T2 hyperintensity. Cortical surface areas also showed FP detections, often attributed to susceptibility artifacts. Additional FP sources included pathologic T2 hyperintensity from subacute infarction or small vessel disease (23), and susceptibility artifacts from cerebral microbleeds. Representative FP cases are shown in Figure 5.

**Table 4:** Location and mechanism of ai false positive lesions.

Location	Interface SA^*^	Intrinsic T2H^†^	Pathologic T2H^‡^	Pathologic SA^§^	Indeterminate	Total
**Cerebral cortex**	66 [20.6]	13 [4.0]	2 [0.6]	10 [3.1]	2 [0.6]	**93 [29.0]**
**Internal capsule**	0 [0.0]	71 [22.1]	0 [0.0]	0 [0.0]	0 [0.0]	**71 [22.1]**
**Pons**	13 [4.0]	4 [1.2]	26 [8.1]	3 [0.9]	0 [0.0]	**46 [14.3]**
**Cerebellum**	35 [10.9]	1 [0.3]	0 [0.0]	2 [0.6]	0 [0.0]	**38 [11.8]**
**PVWM**	0 [0.0]	4 [1.2]	14 [4.4]	10 [3.1]	0 [0.0]	**28 [8.7]**
**Medulla**	11 [3.4]	5 [1.6]	0 [0.0]	1 [0.3]	0 [0.0]	**17 [5.3]**
**Basal ganglia**	3 [0.9]	2 [0.6]	3 [0.9]	4 [1.2]	1 [0.3]	**13 [4.0]**
**Midbrain**	0 [0.0]	9 [2.8]	0 [0.0]	1 [0.3]	0 [0.0]	**10 [3.1]**
**SCWM**	0 [0.0]	0 [0.0]	3 [0.9]	2 [0.6]	0 [0.0]	**5 [1.6]**
**Total**	**128 [39.8]**	**109 [34.0]**	**48 [15.0]**	**33 [10.3]**	**3 [0.9]**	**321 [100]**

Proportion percentage in square brackets. Values exceeding 10% are underlined. AI, artificial intelligence; SA, susceptibility artifacts; T2H, T2 high signal intensity; PVWM, periventricular white matter; SCWM, subcortical white matter. ^*^False positive lesion caused by susceptibility artifacts at brain/skull or brain/air interfaces. ^†^False positive finding due to accentuated T2 signals of normal anatomical structures at the site. ^‡^False positive due to accentuated T2 signals from adjacent pathologic lesions, such as small vessel disease or focal gliosis. ^§^False positive lesion resulting from susceptibility artifacts caused by adjacent pathologic lesions, such as microbleeds or hemorrhage. Bold values indicate row and column totals.

To quantify false positives that most strongly affected reader decisions, we analyzed AI-induced false-positive calls, defined as cases rated true negative without AI but changed to false positive with AI assistance. This analysis identified 19 misleading AI false-positive marks, most commonly intrinsic T2-high–signal–like false positivity in the internal capsule (7/19, 36.8%), followed by pathologic T2-high–signal–like false positivity in the pons (4/19, 21.1%) (Supplementary Table S3). Among the 42 cases with AI false-positive marks, the proportion of AI false-positive cases that led to a false-positive call varied across readers (9.5%−26.2%; Readers 1–5: 16.7%, 9.5%, 11.9%, 26.2%, and 9.5%, respectively).

### Subgroup analysis

Diagnostic performance was evaluated in both challenging cases and positive control cases to assess the practical benefits of AI. In challenging cases, AI improved sensitivity by 20.9% and DSC by 0.362, surpassing improvements observed in the overall cohort (sensitivity, +16.0%; DSC, +0.219) (*p* < 0.01) (Supplementary Table S4). For positive control cases, all readers achieved 100% sensitivity regardless of AI use. However, for cases that readers identified as positive, the addition of AI significantly improved the spatial overlap between readers' delineations and the ground truth (DSC) for all readers (*p* < 0.01) (Supplementary Table S5).

## Discussion

This study explored the clinical impact of AI for detecting AIS lesions on DWI, with a particular focus in challenging scenarios. With JLK-DWI, readers' overall diagnostic performance improved significantly (AUC: 0.93 vs. 0.85) and lesion segmentation became more accurate (DSC: 0.74 vs. 0.52). While the initial radiology reports demonstrated higher sensitivity than standalone AI (98.4% vs. 96.0%), AI still flagged the majority of cases missed on the initial radiology reports [79.6% (43 of 54)].

Despite DWI's high sensitivity, very small or hyperacute lesions, especially in the brainstem, can be missed (6–9). In our cohort, 25.7% (932 of 3623) of AIS cases and 70.4% (38 of 54) of FN cases in the radiology reports were classified as challenging cases, reflecting the difficulty of detecting such lesions. The difference in sensitivity between challenging and positive control cases among the readers (66.8% vs. 100%) further highlights this challenge. Indeed, AI correctly identified all non-challenging cases (100%) and most challenging FN cases (73.7%), thereby reducing human error and aiding in detecting difficult lesions. Theoretically, if all FN cases had been identified and accepted, sensitivity would have increased from 98.4 to 99.7%. Given the already high accuracy of radiology reports, this represents a notable improvement.

The modest standalone specificity of JLK-DWI reflects an inherent trade-off of its DWI-only analysis, which prioritizes maximizing sensitivity to detect potential lesions (24, 25). While incorporating ADC maps could improve specificity, ADC is generally reliable in the hyperacute/acute window; however, in very early presentations and/or very small infarcts, ADC abnormalities may be subtle or spatially heterogeneous, which can limit visual conspicuity on ADC maps in some cases (26–29). Accordingly, our workflow presented the corresponding ADC maps alongside AI outputs for readers' final judgment. We found that most of the AI's false-positive marks stemmed from common mimics such as susceptibility-related effects or T2 shine-through artifacts (Table 4, Figure 5). In addition, our analysis of misleading false-positive marks—those that prompted readers to change an otherwise correct negative judgment to a false-positive call—suggests that anatomically plausible appearances/locations may be more likely to mislead readers. While interface susceptibility artifacts accounted for a large proportion of standalone false-positive marks (39.9% of standalone false positives), they were rarely misleading. In contrast, intrinsic T2-high–signal–like patterns in typical lacunar-infarct locations, including the internal capsule (36.8% of misleading false positives) and pons (21.1% of misleading false positives), were more clinically consequential mimics (Supplementary Table S3). Given that these locations are common sites of lacunar infarction, these findings may inform future refinement efforts aimed at reducing clinically consequential false-positive marks while preserving a sensitivity-prioritizing operating point. Overall, readers were generally able to override these suggestions, resulting in only a modest reduction in overall specificity, although a subset did mislead readers.

Furthermore, readers' confidence scores increased in challenging cases, suggesting that AI could help alleviate the mental stress associated with difficult cases. Even in cases that readers had already identified correctly, lesion segmentation accuracy was significantly improved with the assistance of AI (Supplementary Table S5). The observed increase in DSC suggests that readers either identified more AIS lesions or delineated them more precisely. Given that infarction size influences treatment decisions and outcomes (30), this improvement is clinically meaningful. Unlike radiologists, who have access to electronic medical records which provide useful clinical information, JLK-DWI relies solely on imaging data—underscoring its strong performance.

While many AI models are benchmarked for standalone accuracy (31), our findings address a more critical question: whether integrating AI as a collaborative tool enhances clinical practice. The true value of the AI model was not in its standalone performance, but in its robustness when faced with difficulty. While the accuracy of the initial radiology reports understandably declined for challenging cases, the AI's performance remained stable, highlighting its utility in these scenarios (Table 2).

This stability creates a powerful synergy. Readers leveraged the AI's consistent, high sensitivity to identify subtle lesions, while using their clinical judgment to override the AI's FPs. However, the low standalone specificity of the system (51.8%) introduces the risk of 'alarm fatigue' in high-volume emergency settings. Continually adjudicating false-positive marks imposes a cognitive and temporal burden on readers, exacerbating the 'physician's superhuman dilemma'—the expectation to perfectly calibrate reliance on AI without increasing burnout (32), Although AI standalone specificity is at the lower end of the current spectrum (33), this further reinforces our main conclusion: its primary clinical value lies in augmenting, not replacing, human expertise. Ultimately, our results suggest that even an AI with limitations can meaningfully enhance patient care by providing a stable, sensitive second look where it is needed most.

This study has some limitations. First, as a retrospective single-center analysis based on a stroke registry, our findings may not fully generalize to broader emergency department populations, and the cohort may reflect local referral patterns and relatively homogeneous patient characteristics. In addition, device and acquisition heterogeneity was only partially captured within a single-center setting, and some registry-derived clinical variables (particularly symptom onset time and documentation) may be subject to uncertainty, which could influence time-based subgroup definitions. More broadly, prior methodological work has highlighted that heterogeneity and uncertainty in real-world clinical data collection can propagate downstream uncertainty in medical decision systems, underscoring the need for standardized prospective data capture in future multicenter validation studies (34). Second, our reader pool consisted primarily of junior trainees, which likely magnified the observed diagnostic gains. In real-world workflows, baseline reader sensitivity differs; for senior neuroradiologists in high-volume centers, the absolute diagnostic gain would likely be smaller, and the AI might serve more as a ‘safety net' against rare misses during busy shifts rather than a primary diagnostic aid. Readers were also blinded to clinical information (including presenting symptoms and onset-to-imaging time) and interpreted scans without supervision, which does not fully reflect routine practice; accordingly, baseline reader sensitivity and the magnitude of AI benefit may differ in real-world workflows where clinical context and expert consultation can influence interpretation. Furthermore, “challenging cases” were defined strictly by DWI-based detectability (small-volume/early infarcts) and did not encompass broader clinically challenging dimensions (e.g., complex multifocal presentations or artifact-dominant examinations). Third, our reliance on standard 5-mm thick slices with a 1-mm gap to establish ground truth may have reduced volumetric precision due to partial volume averaging, potentially missing microscopic lesions. Consequently, the true incidence of ultra-small challenging lesions may be underrepresented. Future prospective studies are needed to evaluate challenging strokes using high-resolution, thin-section DWI. Finally, interpretation time was not measured, and therefore we could not quantify the time required to adjudicate false-positive marks. Future prospective multicenter studies should measure interpretation time and evaluate whether AI assistance introduces time penalties or improves overall workflow efficiency; additional work may explore strategies to reduce false positives and mitigate potential alarm fatigue.

In conclusion, JLK-DWI significantly enhanced readers' diagnostic performance for detecting acute ischemic stroke, particularly in challenging cases involving hyperacute or small posterior circulation infarcts. The AI system maintained high sensitivity and provided reliable lesion localization, allowing readers to better identify subtle or easily missed lesions while using their clinical judgment to minimize false positives.

These findings highlight AI's complementary role as an assistive tool that strengthens radiologists' diagnostic accuracy rather than replacing human expertise. Its consistent performance in difficult diagnostic settings suggests potential value for real-world stroke care, supporting faster and more confident decision-making in the acute phase.

## Data Availability

The raw data supporting the conclusions of this article will be made available by the authors, without undue reservation.
