# Supplementary material for: Artificial intelligence–assisted detection of challenging ischemic stroke on diffusion-weighted imaging: a reader study
Source: Front Neurol. 2026 May 21;17:1766199. doi: 10.3389/fneur.2026.1766199 (PMC13233238; doi:10.3389/fneur.2026.1766199)

**Table S1. Distribution of NIHSS scores at admission in the challenging cases**

| NIHSS Category | No. of patients |
|---|---|
| **No stroke symptoms (0)** | 187 (20.1%) |
| **Minor stroke (1–4)** | 499 (53.5%) |
| **Moderate stroke (5–15)** | 220 (23.6%) |
| **Moderate to severe stroke (16–20)** | 17 (1.8%) |
| **Severe stroke (21–42)** | 9 (1.0%) |
| **Total** | 932 |

Note.— NIHSS = National Institutes of Health Stroke Scale. Categories were defined as follows: no stroke symptoms (0), minor stroke (1–4), moderate stroke (5–15), moderate to severe stroke (16–20), and severe stroke (21–42). Percentages are calculated out of the 932 patients classified as challenging cases.

**Table S2. Standalone AI sensitivity by TOAST stroke etiology (top three categories)**

| TOAST etiology | No. of AIS | True positive | False negative | Sensitivity (%) |
|---|---|---|---|---|
| **Large-artery atherosclerosis** | 1506 (41.6%) | 1456 | 50 | 96.7 (95.6–97.5) |
| **Cardioembolism** | 752 (20.8%) | 730 | 22 | 97.1 (95.6–98.1) |
| **Small-vessel occlusion** | 598 (16.5%) | 576 | 22 | 96.3 (94.5–97.6) |

Note.—Data in parentheses are 95% confidence intervals. The between-group difference in sensitivity across the three etiologies was not significant ($p = 0.740$, chi-square test of equal proportions).

**Table S3. Location and presumed mechanism of AI false-positive marks that misled readers**

| | **Interface SA*** | **Intrinsic T2H†** | **Pathologic T2H‡** | **Pathologic SA§** | **Total** |
|---|---|---|---|---|---|
| Cerebral Cortex | 0 [0.0] | 0 [0.0] | 0 [0.0] | 1 [5.3] | 1 [5.3] |
| Internal Capsule | 0 [0.0] | <u>7 [36.8]</u> | 0 [0.0] | 0 [0.0] | 7 [36.8] |
| Pons | 0 [0.0] | <u>2 [10.5]</u> | <u>4 [21.1]</u> | 0 [0.0] | 6 [31.6] |
| Cerebellum | 1 [5.3] | 1 [5.3] | 0 [0.0] | 0 [0.0] | 2 [10.5] |
| Basal Ganglia | 0 [0.0] | 0 [0.0] | 1 [5.3] | 0 [0.0] | 1 [5.3] |
| Midbrain | 0 [0.0] | <u>2 [10.5]</u> | 0 [0.0] | 0 [0.0] | 2 [10.5] |
| **Total** | 1 [5.3] | 12 [63.2] | 5 [26.3] | 1 [5.3] | 19 [100] |

Note.—Proportion percentage in square brackets. Values exceeding 10% are underlined. AI = artificial intelligence, SA = susceptibility artifacts, T2H = T2 high signal intensity, *False positive lesion caused by susceptibility artifacts at brain/skull or brain/air interfaces. †False positive finding due to accentuated T2 signals of normal anatomical structures at the site. ‡False positive due to accentuated T2 signals from adjacent pathologic lesions, such as small vessel disease or focal gliosis. §False positive lesion resulting from susceptibility artifacts caused by adjacent pathologic lesions, such as microbleeds or hemorrhage.

**Table S4. Diagnostic Performance of Readers for Challenging Cases**

| | With AI | Without AI | Difference | P-value |
|---|---|---|---|---|
| **Sensitivity (%)** | | | | |
| **All readers** | **87.7 (85.2–90.0)** | **66.8 (63.1–70.4)** | **20.9** | **< 0.01** |
| **Reader 1** | 84.6 (78.4–90.8) | 62.3 (54.0–70.6) | 22.3 | < 0.01 |
| **Reader 2** | 86.2 (80.2–92.1) | 77.7 (70.5–84.8) | 21.5 | < 0.01 |
| **Reader 3** | 80.0 (73.1–86.9) | 58.5 (50.0–66.9) | 21.5 | < 0.01 |
| **Reader 4** | 94.6 (90.7–98.5) | 83.8 (77.5–90.2) | 10.8 | < 0.01 |
| **Reader 5** | 93.1 (88.7–97.4) | 51.5 (42.9–60.1) | 41.5 | < 0.01 |
| **DSC** | | | | |
| **All readers** | **0.636 (0.610–0.663)** | **0.274 (0.253–0.295)** | **0.362** | **< 0.01** |
| **Reader 1** | 0.679 (0.619–0.739) | 0.351 (0.298–0.405) | 0.178 | < 0.01 |
| **Reader 2** | 0.629 (0.569–0.690) | 0.158 (0.130–0.185) | 0.365 | < 0.01 |
| **Reader 3** | 0.640 (0.576–0.703) | 0.251 (0.206–0.296) | 0.221 | < 0.01 |
| **Reader 4** | 0.492 (0.446–0.538) | 0.393 (0.345–0.441) | 0.016 | < 0.01 |
| **Reader 5** | 0.743 (0.689–0.796) | 0.217 (0.175–0.260) | 0.313 | < 0.01 |

Note.—Data in parentheses are 95% confidence intervals. Bold text highlights overall performance across all readers. AI = artificial intelligence, DSC = Dice similarity coefficient.

**Table S5. Dice Similarity Coefficient (DSC) for Reader-identified Positive Cases**

| | With AI | Without AI | Difference | P-value |
|---|---|---|---|---|
| **All readers** | **0.776 (0.758–0.793)** | **0.473 (0.454–0.492)** | **0.303** | **< 0.01** |
| **Reader 1** | 0.846 (0.815–0.878) | 0.631 (0.598–0.665) | 0.215 | < 0.01 |
| **Reader 2** | 0.793 (0.753–0.834) | 0.222 (0.198–0.246) | 0.571 | < 0.01 |
| **Reader 3** | 0.843 (0.811–0.874) | 0.526 (0.487–0.566) | 0.316 | < 0.01 |
| **Reader 4** | 0.579 (0.540–0.618) | 0.519 (0.481–0.556) | 0.060 | 0.03 |
| **Reader 5** | 0.832 (0.796–0.868) | 0.502 (0.467–0.537) | 0.330 | < 0.01 |

Note.—Data in parentheses are 95% confidence intervals. Bold text highlights overall performance across all readers. AI = artificial intelligence.

**Figure S1. Distribution of pre-stroke and 3-month post-stroke modified Rankin Scale (mRS) scores in the challenging case cohort.**

The bars indicate the percentage of patients at each mRS score (0 = no symptoms to 6 = death) among 932 patients in the challenging case cohort.

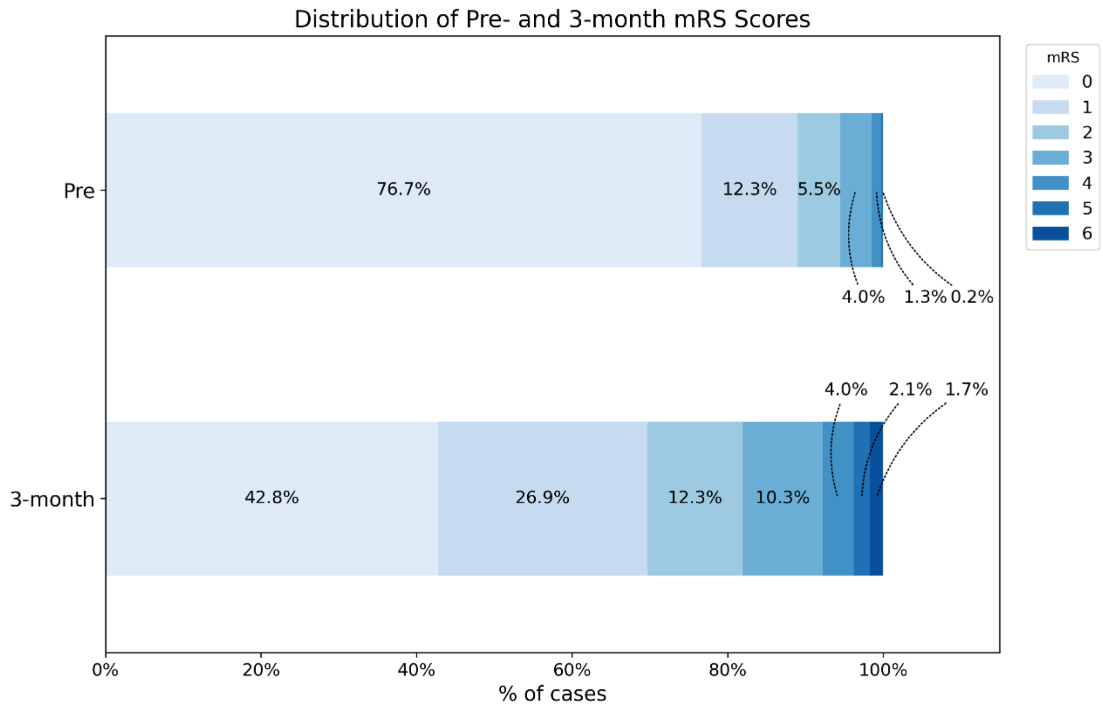

**Figure S2. Comparison of confidence scores with and without AI assistance.**

Violin plots depicting the distribution of confidence scores for positive control (red), challenging cases (green), and negative control (blue), both with and without AI assistance. AI assistance demonstrates an increase in confidence scores, particularly in challenging cases

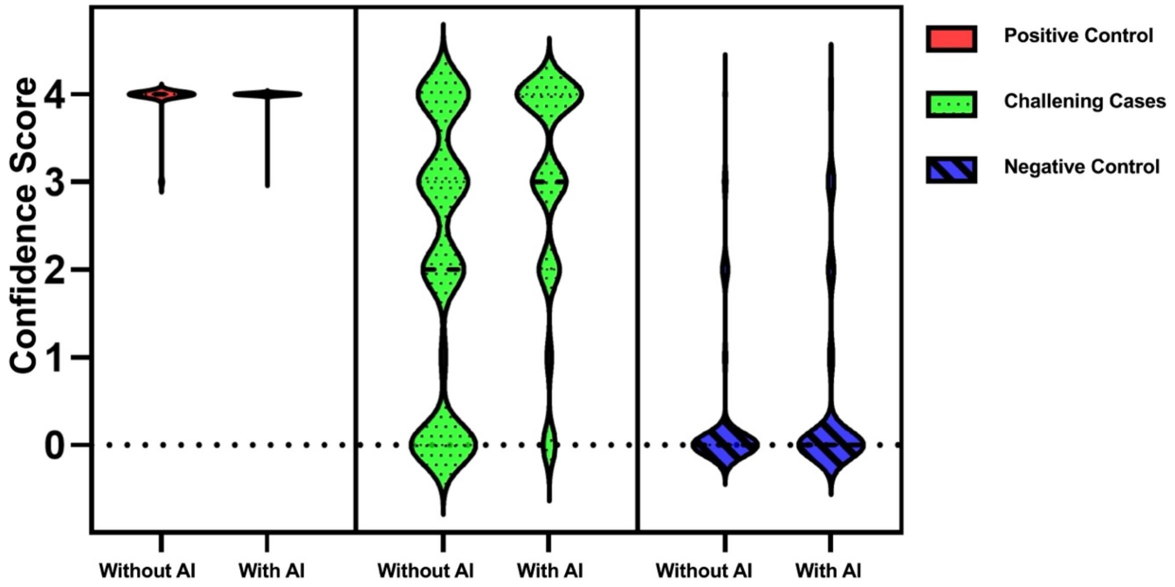

Supplement: Supplementary file 1 [file Data_Sheet_1.pdf]
